# Proper partograph utilization among skilled birth attendants in Hawassa city public health facilities, Sidama region, Ethiopia, in 2021

**DOI:** 10.1186/s12905-022-02117-x

**Published:** 2022-12-22

**Authors:** Berhan Tsegaye Negash, Yitateku Alelgn

**Affiliations:** Department of Midwifery Collage of Medicine and Health Science, Hawassa University, Hawassa, Ethiopia

**Keywords:** Proper, Parthograph, Utilization, Ethiopia

## Abstract

Abnormalities of labor are the major causes of maternal and fetal mortality and morbidity. Proper partograph utilization is a key intervention to detect labor abnormalities and subsequent initiation of management. Although a great deals of studies were conducted about partograph utilization, they have failed to explore some critical factors which correlate with correct filling of partograph so far. To assess magnitude and factors associated with proper partograph recording among skilled delivery attendants in public health facilities of Hawassa city, Sidama Ethiopia, in 2021. An institution based cross-sectional study was conducted to assess proper partograph filling practice among skilled delivery providers of public health facilities of Hawassa city, Sidama region, Ethiopia from November to December 15, in 2021. Data were collected using self-administered questionnaire, and client chart review. Data were entered, cleaned, and analyzed using SPSS software. Binary and multivariate logistic regression analysis was used to show association between outcome and explanatory variables. Multi-collinearity test was done using VIF. Adjusted Odds Ratio with 95% CI and *p* value less than 0.05 was taken as cuff of value for statistically significant value at final model. Out of 405 study participants, only 370 study subjects have provided full response for questions making a response rate of 91.4% in this study. The reason of non-responders was evaluated as not related with the issue of the outcome variable. The mean age of study subjects was 28 ± 3.9 years. Magnitude of proper partograph utilization was found to be58.4% (95% CI, 55.8–60.9%) among skilled delivery attendants in this study. Factors associated with partograph uptake were: On job training (AOR = 1.9, 95% CI: (1.1, 3.2), good knowledge (AOR = 3.1, 95% CI: (1.8, 5.3) and supportive supervision (AOR = 4.5, 95% CI, 2.5, 7.9), client took Uterotonics (AOR = 2.3, 95% CI: 1.4, 3.9), and day time admission (AOR = 3.5, 95% CI, 1.9–6.4). These factors were associated positively with proper partograph utilization. In conclusion, magnitude of proper partograph utilization was found to be lower than magnitude of WHO threshold. Hence, on job training should be enhanced about proper partograph utilization. Furthermore, monitoring, supervision and strengthening the human resource of delivery process would be mandatory by managers of delivery units.

## Background

Globally, 295,000 women died due to childbirth complications in 2016. Most of these deaths (99%) occurred in developing countries. However, they could be prevented using different strategies including partograph utilization [1]. Although many maternal mortalities can be prevented by using partograph properly, one out of ten women still die due lack of proper diagnosis and management [2–6].

Partograph is a simple tool used to recognize emerging complications and manage with standard clinical management protocol [7]. It is one of the leading quality indicator in delivery service [8]. Partograph is a graphic representation of process of labor and delivery. It can display components of labor progress, fetal and maternal wellbeing [9]. Specifically, the labor progress component includes uterine contraction, cervical dilation, and descent of the presenting part. Moreover, the status of fetal condition consists of fetal heartbeat, amniotic fluid volume and molding. Finally, the status of maternal condition consists of the following clinical parameters: Pulse rate, blood pressure, temperature, and urine for volume, protein, and ketone bodies [10]. A partograph has clear demarcations, if arrived at or exceeded clearly indicate the need to address existing or imminent complications [11].

According to national protocol of Ethiopia, skilled delivery attendants should monitor childbirth process by recording foetal, maternal and labour progress on the partograph [5]. The main purpose of partograph is identification of fetal and maternal complications in appropriate time [10, 11]. It is an early warning sign for any fatal condition [12, 13].

The lack of partograph use may result in poor identification of intrapartum complication and delayed intervention [14]. Previous evidence suggested that uterine rupture is associated with failure to use partograph properly [15, 16]. Furthermore, another study also indicated that partograph utilization decreases cesarean delivery rate [17–19]. Partograph as a referral decision making tool by health workers in primary level care facilities [20]. Previous study showed that women who obtained less person-centered care at night time [21]. Previous study finding indicated that clients are strictly followed when they are induced or augmented using Uterotonics drug [22].

Adequate knowledge about partograph use, positive attitude for utilization of partograph and on job training about partograph uptake was found influencing positively for partograph utilization [23–25]. A study conducted in Addis Ababa Ethiopia shows that skilled delivery attendants working in health centres more utilize partograph than hospitals [14]. Another study also indicate that type of profession and sex of the skilled providers were associated with proper partograph utilization [3]. A study conducted in west Shoa zone, central Ethiopia indicated that the presence of supportive supervision for skilled delivery attendants was positively associated with proper partograph utilization, adequate number of skilled providers can increase the chance of partograph utilization, and presence of standard protocol in the health facility were associated with partograph utilization [25]. Although partograph is cost effective and technically simple that can be used by all level of skilled delivery attendants, little attention have been granted for its implementation in most resource poor settings [23, 26]. The World Health Organization (WHO) recommends using the partograph to follow labour and delivery, with the objectives to improve health care and reduce maternal and foetal morbidity and death [27]. Despite, perinatal mortality can be prevented using simple cost-effective antenatal and intrapartum interventions as antenatal care visit and partograph utilization the rate of perinatal mortality remains a major challenge in Ethiopia [28, 29]. Although many studies were conducted previously in Ethiopia, they have missed some critical factors associated with practice of partograph filling: Time of client admission and use of Uterotonics during child birth. Furthermore, these studies failed to measure the proper partograph utilization using a process based composite variable. Measuring partograph utilization by simple ‘yes’ and ‘no’ question will loss much information.

Therefore, this study aimed to identify magnitude and factors associated with proper partograph utilization among skilled delivery attendants in public health institutions of Hawassa city, Sidama regional state Ethiopia, in 2021.

## Methods

### Study setting

This study was conducted in public health facilities in Hawassa city administration. Hawassa city is located 275 km to the South of Addis Ababa, the capital city of Ethiopia [30]. Hawassa city administration has 28 public health institutions. There are a total of 3 hospitals: One comprehensive specialized referral hospital, 1 general hospital, 1 primary hospital, 11 public health centers, and 17 health posts with functional delivery units. There were 480 skilled delivery attendants who provide delivery service in public health institutions of Hawassa city during the study period.

### Study design and period

This analytical cross sectional study was designed to explore magnitude and factors associated with proper partograph utilization among skilled delivery attendants in Hawassa city public health facilities, Sidama region, Ethiopia. This study was conducted from November 15 to December 15, in 2021.

### Population

All skilled delivery providers who attended delivery at child birth units of public health facilities of Hawassa city were the source population. On the other hand, all skilled delivery attendants who conducted delivery service in the selected health institutions, and presented during data collection period were the study population in this study. However, study subjects who were on sick leave, annual vacation and unable to provide full response due to serous sickness were excluded from this study.

### Sample size determination and sampling procedure

In this study, sample size was determined by using single population proportion formula with the following assumptions: Prevalence of proper partograph utilization was taken as 54.4% from previous study done in North Shoa, Ethiopia [23], 95% Confidence Interval, and margin of error as 0.05. Cochran’s standardized formula was used to compute the minimum adequate sample size to attain objectives of this stud. Thus, the sample size used to determine the prevalence of partograph utilization (first objective) was found maximum than sample sizes calculated to explore associated factors associated with partograph utilization (second objectives). Therefore, using the following formula: n = Z a/_2_)^2^p (1 − p)/d^2^, we have calculated initial sample size as 368. Where, n; sample size, P; magnitude of proper partograph utilization from previous study, Z = significance level of standard normal value which corresponds with 95% (1.96). We plug in the values into formula and computed as, n = 1.96^2^ × 0.4(1–0.6)/0.05 = 368. Then, 10% non-response rate was added and got a final sample size of 405.

In this study, three hospitals: Hawassa university comprehensive specialized hospital, Adare general hospital and Mutitie fura district hospital; and five health centers (30–40% of the total health centers) were randomly selected and included in this study. Finally, all skilled delivery attendants in the selected health facilities were included in the study.

### Data collection strategy, tools and procedure

In this study, we have utilized triangulated data collection strategy to gather information from the study participants. Self-administered questionnaire and client chart review were conducted concurrently for each study participants. The questionnaire contains information about socio-demographic factors, knowledge, and attitude. Furthermore, client chart review was performed using structured checklist to seek clinical factors and outcome variable. The reliability and validity of the questionnaire and checklist were ensured through adequate literature review [4, 5], expert review. Furthermore, pretest was done among 5% of the total sample size in an area other than the study area, but with similar set up. The purpose of the pretest was to ascertain problems with data collection tool and to find solutions. We have employed 3 degree midwives as data collectors and 2 MSc clinical midwives as supervisors. Data quality was assured by using standard data collection tool, recruitment of qualified data collectors, intensive supervision. Besides, mechanisms to minimize information contamination were put in place. For example, we recruit data collectors from health facilities outside of the study setting.

### Study variables

The dependent variable for this study was proper partograph utilization. It was the binary outcome variable. In this specific study, proper partograph utilization was constructed as a composite variable which consists of 10 sub-components. Each sub-component was further sub-divided into specific partograph components. These include the following main and subcomponents: Maternal condition component consists of 4 sub-components, labour progress consists of 3 sub-components, and foetal condition comprises 3 sub-components. Each sub-component was labelled as ‘yes’ or ‘no’ for the ‘performance’ and ‘non-performance’ of the components respectively. Hence, ‘yes’ answer was coded as ‘1’, otherwise, ‘0’. Finally, we have computed the total score and dichotomized as ‘1’ and ‘0’ for practice and non-practice of partograph properly.

In this study, the explanatory variables included in this study were socio-demographic factors like: Age, sex, qualification, profession, place of work and service year. Furthermore, the health behaviour characteristics of the skilled delivery attendants included: Knowledge, attitude, on job training and supportive supervision. Attitude and knowledge were also composite variables measured indirectly by asking them sub-component questions: The knowledge of proper partograph was assessed by asking 10 questions. Each question was designed as close ended questions with binary possible answers, either ‘yes’ or ‘no’. Hence, the sum of the scores of the individual case was further dichotomized and recoded as ‘1’ for adequate knowledge if the score is greater than 5 (mean score), otherwise, ‘0’. Furthermore, attitude of the study participants was highlighted by using 8 different questions. Hence, study subjects were asked to score either ‘1’ or ‘0’ for agree or disagree respectively. Finally, the total score was calculated and re-categorized again for analysis. The total score more than 4 or more was labelled as ‘positive’ and coded as ‘1’, and the total score less than 4 was labelled ‘negative’ and coded as ‘0’.

### Data quality control

The principal investigator has prepared questionnaire by reviewing different literatures. The questionnaire was prepared in English then translated to Amharic by an expert. The tool was retranslated back to English to maintain its consistency. It was pretested pre-tested at Yirgalem General Hospital which is 60 km from the study area at 5% of total samples. Observation was made till the end of delivery. Observation checklist was developed as a tool from the partograph chart for direct observation of study participants during the progress of labour. All data collectors and supervisors were recruited outside of the study setting. They were trained for three consecutive days about objective of the study, technique of data collection, components of questionnaire and components of checklist. All data were registered and checked for completeness immediately after data collection by data collectors and supervisors. Data collectors distributed the questionnaire to midwives who were selected as a sample after explaining the purpose of the study and obtaining written informed consent.

### Data processing and analysis

The data was entered, cleaned and analyzed using SPSS version 26. Descriptive analysis was made using percentage, mean and standard deviation for variables included in the study. Frequencies was first listed and followed by cross tabulation to compare among frequencies. Both bivariable and multivariable logistic regression analysis were performed to determine the association of independent and dependent variables. The necessary logistic regression assumptions were checked and fixed before the actual data analysis. Chi-square test of association was conducted for categorical variables before bi-variable logistic regression analysis. Variables whose *p* value less than 0.05 were further tested using bi-variable logistic regression analysis. In bivariable logistic regression analysis, variables with a *p* value < 0.25 were fitted into multivariable logistic regression analysis model to adjust for the effect of confounders. In multivariable logistic regression analysis model, Odds Ratios (ORs) with 95% confidence intervals were reported to show the presence of statistically significant associations. Statistical significance was declared at *p* < 0.05. Hosmer and Lemeshow goodness of fit was used to check the model fitness and found *p* value less than 0.05. Tables were used to present the findings of this study.

### Operational definitions

#### Practice of proper partograph utilization

Proper partograph utilization was a composite variable which consists of 10 checklists; each of which has binary outcome. The presence of proper partograph utilization was declared using the sum scores of the total components. a score of 6 and above was categorized as ‘adequate’, otherwise, inadequate’ practice of partograph [4, 23].

#### Knowledge of partograph

Knowledge was measured using ten questions. Those who responded six (60%) questions as ‘yes’ were labelled as having ‘adequate’,otherwise, ‘inadequate’ knowledge in the final analysis [5].

#### Attitude towards partograph use

Those study subjects who select ‘agree’ at least five attitude questions were categorized as ‘positive’, otherwise, ‘negative’ and coded as ‘1’ and ‘0’ respectively.

## Result

### Descriptive analysis of socio-demographic characteristics

From the total of 405 samples, only 370 study subjects provided complete response making a response rate of 91.3%. This interruption of the questionnaire by study subjects was proved to be random than intentional. The mean age of the study students was 28.7 years with SD ± 3.9 years. Table 1 summarizes the socio-demographic variables of the study subjects in this study. Accordingly, most (60.5%) of the study subjects participated in this study were females. Majorities (72.4%) of the study subjects were midwives. Almost one out of four (26.4%) of the study subjects acquired supportive supervision (see Table 1).

**Table 1:** Socio-demographic and health behavioral characteristics of skilled delivery attendants in Hawassa city health facilities, South Ethiopia, in 2021 (n = 370)

Variable	Category	Number	Percent (%)
Age in years	20–29	237	64.1
	30–39	127	34.3
	40 and above	6	1.6
Sex	Female	224	60.5
	Male	146	39.5
Qualification	Diploma	120	32.4
	Bachelor degree	236	63.8
	Master degree	14	3.8
Profession	Midwifery	274	72.4
	Nurse	74	20
	Public health officer	22	5.9
Pace of work	Hospital	223	60.3
	Health center	147	39.7
Service year	Less than 4	119	32.2
	04-Jun	126	34.1
	7 and above	125	33.8
On job training on Partograph	Yes	243	65.7
	No	127	34.3
Attitude	Positive	60	12.6
	Negative	322	84.4
Supportive supervision	Yes	98	26.4
	No	272	73.6

### Knowledge of proper partograph utilization

Table 2 enumerates the components of proper partograph utilization. Hence, only sixty point eight percent (60.8%) of the study subjects knew how to fill partograph properly. Effective standard for observing progress of labour (93.2%) was the most known component of partograph. In contrast, only fifteen point one (15.1%) of the study subjects knew that partograph utilization can allow time for further discussion of management (see Table 2).

**Table 2:** Knowledge of proper parthograph uptake among skilled delivery attendants in Hawassa city health institution, South Ethiopia, in 2020 (n = 370)

Advantages of proper parthograph utilization	Count	Percent
Early prediction of deviation from normal progress of labour	354	92.7
Alert birth attendants	312	81.7
Reduce morbidity and mortality	351	91.9
Simple technique	267	69.9
Provides information on single sheet of paper at a glance	144	39.9
Low cost	234	62.2
Easily accessible	261	70.5
Effective standard for observing the progress of labor	345	93.2
Can allow time for further discussion of management	56	15.1
Make observation and recording more objective	123	33.2
Identify necessary intervention	321	86.7
Help to make quick and logical decision for managing labor	312	84.3

### Practice of proper partograph utilization

Table 3 depicts the magnitude of proper partograph uptake in this study. It displays that more than half (58.4%) of skilled delivery attendants utilized partograph properly. The maternal condition component of the partograph was the more ignored component compared to the other two components of the Partograph. Out of the 370 reviewed Partograph charts, only 39 delivering mothers’ urine samples were checked for volume, protein and ketone bodies. From the total reviewed charts 348 (94.1%) had cervical dilation recorded, 222 (60%) recorded descent, 340 (91.9%) had plotted uterine contraction, 298 (80.5%) registered fetal heart rate, and 60% of the skilled providers plot descent on the partograph. Besides, only one-third and one tenth study subjects record maternal temperature and urine analysis. On the contrary, cervical dilation (94.1%) and uterine contraction (91.9%) are the two main components of labor progress parameter which were recorded more frequently (see Table 3).

**Table 3:** Practice of proper parthograph utilization among skilled birth attendants in Hawassa city administration 2020 (n = 370)

Parameters	Category	Frequency	Percent
Progress of labour			
Plot cervical dilation every 4 h	Yes	348	94.1
	No	22	5.9
Plot descent	Yes	222	60
	No	148	40
Plot uterine contraction every 30 min	Yes	340	91.9
	No	30	8.1
Fetal condition			
Monitor and plot fetal heart rate every 30 min	Yes	298	80.5
	No	72	19.5
Record color of liquor during every per vaginal examination	Yes	198	53.5
	No	172	46.5
Plot molding of fetal skull	Yes	185	50
	No	185	50
Maternal condition			
Plot maternal pulse rate every 30 min	Yes	227	61.4
	No	143	38.6
Plot maternal blood pressure every 4 h	Yes	214	57.8
	No	156	42.2
Plot maternal temperature every 2 h	Yes	104	28.1
	No	266	71.9
Record urine volume, urine protein and ketone every 2–4 h	Yes	39	10.5
	No	331	89.5

Table 4 summarizes the cross tabulation of explanatory variables and outcome variable. Accordingly, type of profession, on job training and knowledge about proper partograph utilization were factors significantly associated with proper partograph uptake in Chi-square test of association (see Table 4).

**Table 4:** Cross tabulation of demographic factors with practice of proper parthograph utilization among skilled care providers in Hawassa city health facilities in 2022 (n = 370)

Variables	Practice of proper parthograph		Pearson Chi-square test value	*p* value
	Yes	No		
Age				
Less than 30 years	93	134	0.24	**0.880**
More than 30 years	61	82		
Sex				
Male	79	111	0.000	**0.980**
Female	75	105		
Service years				
Less than three years	53	66	1.09	**0.570**
Four to seven years	48	78		
More than seven years	53	72		
Profession*				
Midwifery	96	178	19.25	**0.000**
Nurse	46	28		
Health officer	12	10		
Level of education				
Diploma	56	64	2.5	**0.280**
BSC	94	142		
MSc	4	10		
Place of work				
Health centre	58	89	0.47	**0.490**
Hospital	98	127		
On job training*				
Yes	55	38	15.6	**0.000**
No	99	178		
Knowledge				
Adequate	73	61	14.3	**0.000**
In adequate	81	155		

*Pearson Chi-square test-value < 0.005

In cross tabulation (chi-square test),variables with *p*-values < 0.05 were further fitted into binary logistic regression analysis.

### Factors associated with proper partograph uptake

Table 5 presents factors significantly associated with proper partograph utilization in both binary and multivariable logistic regression analysis. In bivariable logistic regression analysis model, six independent variables showed statistically significant association with proper partograph utilization: Knowledge, attitude, on job training, supportive supervision, time of client admission, and using of Uterotonics medication. Except attitude, other variables were also associated significantly with proper partograph uptake in the multi-variable logistic regression analysis model.

**Table 5:** Report of logistic regression analysis of factors associated with practice of proper partograph utilization among skilled delivery attendants work in labor wards of Hawassa city public health facilities, in 2021 (n = 370)

Independent factors	Practice of proper partograph use			COR 95% CI	AOR 95% CI	*p* value
		Adequate	Inadequate			
Knowledge	Adequate	70	155	1.00	1.00	
	Inadequate	84	61	0.32 (0.21–0.50*	2.1 (1.31–3.51)**	0.002
Attitude	Negative	12	9	1.00	1.00	
	Positive	142	206	0.14 (0.57–0.98)	0.9 (0.32–2.55)	0.82
Supportive supervision	No	91	181	1.00	1.00	
	Yes	63	35	3.5 (2.20–5.8)*	4.5 (2.5–7.9)**	0.000
Training	No	99	178	1.00	1.00	
	Yes	55	38	2.6 (1.6–4.2)*	1.9 (1.1–3.3)**	0.021
Uterotonic drugs	No	80	148	1.00	1.00	
	Yes	74	68	2 (1.3–3.0)*	2.3 (1.4–3.9)**	0.001
Time of admission	Night	36	109	1.00	1.00	
	Day	118	107	3.3 (2.1–5.1)*	3.5 (2.1–6.5)**	0.000

*COR* crude odds ratio, *AOR* adjusted odds ratio, *p < 0.25; **p < 0.05

Regarding factors associated in multivariable logistic regression analysis, skilled delivery attendants who trained on partograph utilization had 1.9 times more likely to fill partograph properly than their counterparts (AOR = 1.9, 95% CI: 1.1, 3.37). The chance of practicing proper partograph filling among skilled delivery attendants who had knowledge of proper partograph utilization was 2.1 times more than their counterparts (AOR = 2.1, 95% CI: 1.31,3.51). Skilled delivery attendants who had supportive supervision about proper partograph utilization had 4.5 times more odds of practicing partograph uptake properly than their counterparts (AOR = 4.5, 95% CI: 2.6, 7.9). Furthermore, day time of admission of clients, in contrast to night time client admission, had 3.5 folds of odds of practicing proper partograph use (AOR = 3.5, 95% CI, 2.11, 6.1). Compared to skilled delivery attendants who followed their clients who did not take Uterotonics drugs, skilled delivery attendants who followed clients who took Uterotonics drug had 2.3 times more chance of practicing proper partograph use (see Table 5).

## Discussion

In this study, magnitude of proper partograph utilization was found to be as 58.4% (95% CI, 55.8%, and 60.9%) in public health institutions of Hawassa town, Sidama, Ethiopia. This finding lower than WHO (World Health Organization) standard [31], Tigray, North Ethiopia (73.3%), and Wolayta, South Ethiopia (71.7%) [32]. The possible variation might be linked to difference in health institutions’ monitoring and support system, training availability and staff profile. Furthermore, the availability of printed and structured partograph and individual commitments might be increased in these health facilities than the current study area. Previous studies also highlighted the association of availability and utilization of partograph [2, 33]. Furthermore, these inconsistencies might be explained by various reasons: Lack of adequate knowledge, negative attitude of obstetrics care providers, and lack of adequate training about proper partograph utilization. Moreover, lack of necessary resource, staffs, and low educational status of the skilled birth attendant could be the possible alternative explanations for low uptake of proper partograph.

Based on the report of this study, this finding is consistent with average national magnitude of proper partograph utilization in Ethiopia (59.9%) [24]. This might indicate the finding of this study is more reliable estimate than others. The possible rational for similarities could be justified as the similarities in the study population, setting and charactestics of the study participants.

However, the current finding is higher than study findings studies conducted in North Shoa, Amhara region (40.2%) [23], Hadiya (54.4%) [34], Addis Ababa (57.3%) [35], and South West Nigeria (32.3%) [36]. The possible rational behind this difference might be attributed by less client burden in the study area making minimal work stress with adequate number of skilled delivery attendants in the current study compared with the others. Previous studies mentioned that skilled delivery attendants on duty are overwhelmed with so much work on a shift such that little or no attention is paid to some important and routine professional details [2, 37].

Previous studies also mentioned that health institutions with adequate number of skilled delivery attendant staff utilized partograph than their counterparts [2, 33]. Moreover, the variation might be associated with difference in awareness and attitude among skilled delivery attendants towards proper partograph utilization. Previous studies also revealed existence of significant association between participants’ attitude and knowledge towards the partograph and utilization of it [3, 14].

Based on the finding of this study, skilled delivery attendants who had ever trained about partograph were more likely to utilize partograph properly than their counterparts. This finding is consistent with the findings of study in Ethiopia [24], North Shoa [38], East Gojam [39], Hadiya, Southern Ethiopia [34]. This similarity could be explained by the fact that skilled delivery attendants who had received on the job training on proper partograph utilization might have better knowledge and skill than their counterparts.

Skilled delivery attendants who had supportive supervision from managers are more likely to fill partograph properly than their counterparts. This finding is in line with findings of a studies done in Nigeria [4], Ethiopia [24] and in Addis Ababa, Ethiopia [40]. This might be explained by the fact that at the time of supervision obstetric care givers are motivated to utilize partograph and because of fear of judgment the external supervisor they may utilize partograph. Skilled delivery attendants who were knowledgeable about the Partograph were about three times more likely to utilize the Partograph than those who were not knowledgeable. This is similar with the study done in North Shoa, Ethiopia, Hadiya zone southern Ethiopia [34] in Asella referral hospital [41]. Having good knowledge about proper partograph use might enhance skilled care provider’s competency to use it effectively. This was shown by previous studies which stated that little or no knowledge remains a significant barrier to its effective utilization [2, 3, 37]. Mothers who were admitted at day time were three times more likely to be attended using partograph than their counterparts. This finding is similar with the study conducted in Tigray, Northern Ethiopia [42–44]. This might be diurnal variation is the most commonly cited reason for poor performance in the night shift. Short circadian rhythms of cortisol secretions could influence mental and physical health. This might be explained by the fact that few man power is assigned at night than day time at delivery unit due to financial scarcity for extra-time payment [21]. Furthermore, previous study recommend the low number of midwives and increased work load can impact on the quality of nursing care [45].

Mothers who were induced and augmented were more than two times more likely followed by partograph than their counterparts. Oxytocin induction was started both ‘too early’ and ‘too late’ in relation to the diagnosis of labour before emerging technology of partograph [46].This may be the fear side effect of Uterotonics on the mother and on the fetus and to know the effect of the drug on the progress of labor.

### Strengths and limitations of the study

This study had the following strengths. First, it highlighted that the magnitude of proper partograph utilization using a standard tool. Second, we have explored some novel critical factors that can be input for clinical practice policy making. Finally, data collected from all skilled providers in the selected health institutions minimizes sampling errors. On the contrary, this study had the following limitations: First, the cross section nature of the study does not establish a causal association between explanatory variables and outcome variable. Next, obstetric care providers in public health centers, private and faith–based health institutions, as well as those in rural public health institutions within the region were not included. Thus, generalization of the findings should be made with caution. Final, there is a tendency for social desirability bias whereby study respondents tend to give answers that they think appropriate.

### Clinical practice implication

This study highlighted the importance of refresher on job training of skilled delivery attendants about proper partograph utilization for stakeholders. Furthermore, the health institutions management is expected to create a support and supervision system for monitoring and evaluation of proper partograph utilization by skilled delivery attendants specifically for delivery attendants at night duty. Finally, strict designing of policy and implementation can be applied on skilled delivery attendants to be following all laboring women with partograph properly. Based on the finding of the current study much work will be done on the attitude and awareness.

### Conclusion and recommendation

To conclude, proper partograph utilization is lower than WHO recommended threshold. Lack of knowledge, on job training, and supportive supervision about partograph was factors strongly associated with proper partograph recording and client follow up during childbirth. Moreover, night admission of labor cases and using Uterotonics were significantly associated with partograph utilization. Therefore, updated training should be enhanced all skilled delivery attendants. Health managers and directors should create monitoring and supervision mechanism through child birth auditing system. Policy makers and curriculum designers should adequately incorporate proper partograph utilization in the pre-service training for all type of skilled delivery attendants.

## Data Availability

We have sent all the available data and we do not want to share the raw data as we are doing related study.

## Abbreviations

KAP: Knowledge Attitude Practice
SPSS: Statistical Package for Social Science
WHO: World Health Organization
IMC: International Midwife Confederation
IRB: Institutional Review Board
